# Trends of Infective Endocarditis at Two Teaching Hospitals: A 12-Year Retrospective Cohort Study in Rio de Janeiro, Brazil

**DOI:** 10.3390/tropicalmed8120516

**Published:** 2023-12-12

**Authors:** Paulo Vieira Damasco, Victor Edgar Fiestas Solórzano, Natália Rodrigues Querido Fortes, Daniel Xavier de Brito Setta, Aloysio Guimaraes da Fonseca, Mario Castro Alvarez Perez, João Carlos Jazbick, Jonathan Gonçalves-Oliveira, Marco Aurélio Pereira Horta, Elba Regina Sampaio de Lemos, Claudio Querido Fortes

**Affiliations:** 1 Escola de Medicina e Cirurgia, Departamento de Doenças Infecciosas e Parasitárias, Universidade do Federal do Estado do Rio de Janeiro—UNIRIO, Rio de Janeiro 20271-062, Brazil; 2 Hospital Universitário Pedro Ernesto, Universidade do Estado do Rio de Janeiro (HUPE/UERJ), Rio de Janeiro 20551-030, Brazil; daniel.setta@hupe.uerj.br (D.X.d.B.S.); aloysio.fonseca@hupe.uerj.br (A.G.d.F.); marioperez@unifeso.edu.br (M.C.A.P.); joao.jazbik@hupe.uerj.br (J.C.J.); 3 Programa de Pós-Graduação em Medicina Tropical, Instituto Oswaldo Cruz (IOC/FIOCRUZ), Rio de Janeiro 21040-900, Brazil; vfiestas@ins.gob.pe; 4 Hospital Universitário Clementino Fraga Filho, Universidade Federal do Rio de Janeiro—UFRJ, Rio de Janeiro 21941-617, Brazil; natalia.fortes@ebserh.gov.br (N.R.Q.F.); cfortes@hucff.ufrj.br (C.Q.F.); 5 Laboratório de Hantaviroses e Rickettsioses, Instituto Oswaldo Cruz (IOC/FIOCRUZ), Rio de Janeiro 21040-900, Brazil; jonathan.goncalves@fiocruz.br (J.G.-O.); marco.horta@fiocruz.br (M.A.P.H.)

**Keywords:** endocarditis, mortality, retrospective cohort, Brazil

## Abstract

Background: Despite advances in diagnosis and treatment, the incidence and mortality of infective endocarditis (IE) have increased in recent decades. Studies on the risk factors for mortality in endocarditis in Latin America are scarce. Methods: This retrospective cohort study included 240 patients diagnosed with IE according to the modified Duke criteria who were admitted to two university hospitals in Rio de Janeiro, Brazil from January 2009 to June 2021. Poisson regression analysis was performed for trend tests. The multivariate Cox proportional hazards model was used to estimate the hazard ratio (HR) of predictors of in-hospital mortality. Findings: The median age was 55 years (IQR: 39–66 years), 57% were male, and 41% had a Charlson comorbidity index (CCI) score > 3. Healthcare-associated infective endocarditis (54%), left-sided native valve IE (77.5%), and staphylococcal IE (26%) predominated. Overall, in-hospital mortality was 45.8%, and mortality was significantly higher in the following patients: aged ≥ 60 years (53%), CCI score ≥ 3 (60%), healthcare-associated infective endocarditis (HAIE) (53%), left-sided IE (51%), and enterococcal IE (67%). Poisson regression analysis showed no trend in in-hospital mortality per year. The adjusted multivariate model determined that age ≥ 60 years was an independent risk factor for in-hospital mortality (HR = 1.9; 95% CI 1.2–3.1; *p* = 0.008). Interpretation: In this 12-year retrospective cohort, there was no evidence of an improvement in survival in patients with IE. Since older age is a risk factor for mortality, consensus is needed for the management of IE in this group of patients.

## 1. Introduction

Infective endocarditis (IE) is an infectious disease that requires a multidisciplinary management team. Endocarditis can affect native valves and intracardiac prosthetic materials (prosthetic valves, annuloplasty, cardiac devices, intracardiac patches, and shunts) [1,2]. Intravenous drug use, prosthetic valves, degenerative valve disease, and intracardiac devices are the main risk factors for IE in developed countries. Additionally, rheumatic heart disease and congenital heart diseases contribute to the incidence of IE in low- and middle-income countries [3,4,5,6].

Many factors affect the outcome of IE, including virulence of the causative microorganism, patient characteristics, presence of underlying disease, surgical indications, timing of surgery, and delays in diagnosis and treatment. Infective endocarditis results from microbial infection of the endothelial surface of the heart and frequently causes debilitating morbidities, such as heart failure, stroke, renal failure, and sepsis [3,4]. The characteristic vegetative lesion consists of a mass of platelets and fibrin to which circulating microorganisms adhere and enmesh in a structured biofilm matrix [3]. According to the International Collaboration on Endocarditis-Prospective Cohort Study, where they studied 2781 adults with definite IE were admitted to 58 hospitals in 25 countries between June 2000 and September 2000, the etiology of IE varies with region, but the leader of etiology of IE is *Staphylococcus aureus* [5].

Regarding the incidence, IE has become a significant challenge for public health, especially in the Western world, where the elderly constitute the most critical group of the population, often with many comorbidities, increasing the risk of bacterial infection, especially *Staphylococcus aureus*, a virulent microorganism that has downgraded the rope of Viridans group Streptococci becoming the main etiologic of IE in the 21st Century [4,5,6,7,8]. The global incidence of IE is between 2 and 12 cases per 100,000 people per year [7].

Before the advent of antimicrobials, the lethality of IE was 94% when the treatment was exclusively clinical [9]. With the enormous advances in antimicrobial therapy associated with surgical procedures, the mortality rate has improved over the last century. However, despite the large number of antibiotics available, therapeutic success is still low, considering mortality rates of 36% in high-income countries and greater than 40% in low- and middle-income countries [10,11,12].

We believe that understanding the local epidemiology and risk factors for in-hospital mortality in patients with IE is important for improving the outcomes of this fatal heart infection. This study aimed to report the demographic, epidemiological, etiologic, and risk factors for in-hospital mortality in a retrospective cohort of 240 patients diagnosed with IE over a period of 12 years at two teaching hospitals located in Rio de Janeiro, Brazil.

## 2. Materials and Methods

### 2.1. Study Setting and Participants

Only two teaching hospitals in Rio de Janeiro have a cardiovascular surgery unit and an IE team. The Hospital Universitário Pedro Ernesto (HUPE) and Hospital Universitário Clementino Fraga Filho (HUCFF), both in Rio de Janeiro municipality, are public medical hospitals that serve as both a tertiary care referral center and a primary and secondary care institution for our public assistance security (Sistema Único de Saúde - SUS). The HUPE and HUCFF with 560 and 350 beds, respectively, provide care for all medical and surgical specialties and sub-specialties. This retrospective cohort study analyzed information from medical records of all hospitalized patients with a diagnosis of IE in both hospitals from January 2009 and June 2021. Sociodemographic variables such as age, sex (based on the biological characteristics reported in the medical records), and preexisting conditions were analyzed. The STROBE checklist for reporting observational studies was used.

### 2.2. Infective Endocarditis Diagnosis

All patients with IE were interviewed by the same infectious disease physician (IDP) during the study period. The STROBE checklist for reporting observational studies was used. The inclusion criteria to select to participate in this study were all patients referred to our hospitals for suspected IE. Patients who did not fulfill the criteria of definite IE by Modified Duke Criteria were excluded. Eleven patients in this cohort were excluded from the analysis due to patient transfer to another hospital or missing data.

#### 2.2.1. Modified Duke Criteria

The major criteria included typical microorganisms consistent with IE from two separate blood cultures: community-acquired enterococci, in the absence of a primary focus, or microorganisms consistent with IE from persistently positive blood cultures or positive blood culture for an organism known to cause IE or for *Coxiella burnetii* antiphase I IgG antibody titer > 1:800 and evidence of endocardial involvement provided by echocardiography. The minor criteria included predisposing factors for IE, temperature > 38.0 °C, vascular and immunological phenomena, and microbiologic evidence of active infection with a known pathogen causing IE. Regarding the Pathological criteria, they were: (1) Microorganisms demonstrated by culture or histological examination of vegetation, vegetation that had embolized, or an intracardiac abscess specimen; and (2) vegetation or intracardiac abscess confirmed by histological examination showing active endocarditis [13]. Healthcare-associated infective endocarditis (HAIE) was considered as either IE-manifested > 48 h after admission to the hospital or IE-acquired in association with a significant invasive procedure performed in the 6 months preceding the following situations: (a) stay and/or treatment in a hospital setting (nosocomial health-associated IE); or (b) extensive outpatient contact with healthcare interventions. Demographic, epidemiological, and clinical data from the patient’s first medical visit to the hospital, blood culture results, echocardiographic findings, cardiac surgical interventions, and outcomes were collected.

#### 2.2.2. Echocardiographic Data

Transthoracic and/or transesophageal echocardiography was performed by the echocardiography service at HUPE and HUCFF. Vivid E95 GE equipment employing transthoracic (25 s) and/or 3-dimensional transesophageal echocardiography (10 mHz) probes were performed at the Echocardiography service of the Hospitals. All echocardiography exams in which IE was suspected were reviewed by an expert in Echocardiography in our associated departments (Internal Medicine, Infectious Diseases, Cardiologic and Cardiovascular).

#### 2.2.3. Microbiological Data

Blood cultures were processed using the BD BACTECTM FX system (BD Diagnostics) in the laboratories of the university hospitals, with at least two aerobic bottles incubated for 5 days (14 days in exceptional cases), and VITEK^®^ 2 system (BioMérieux^®^, Marcy-l’Étoile, France) identification cards (ID) and antibiotic susceptibility testing (AST) cards were used according to the manufacturer’s instructions. In addition to the recovery of fastidious bacteria using immunological and molecular methods, the antimicrobial susceptibility profiles were performed by the disk diffusion method diagnosis of microorganisms. When possible, the minimal inhibitory concentration (MIC) of vancomycin was determined by the E-test and both microdilution for *Enterococcus* spp. and methicillin-resistant *Staphylococcus aureus* (MRSA), respectively. In relation to IE due to *Bartonella* spp. and *Coxiella burnetii*, serum samples were evaluated using an indirect immunofluorescence assay for IgG antibodies against anti-*Bartonella* spp. and anti-*C. burnetii* (phase I and II, PANBIO^®^, Brisbane, AU, Australia), with a titer of ≥800 dilutions as the cut-off for positive samples. Afterward, blood and heart valve tissue samples were analyzed using polymerase chain reaction (PCR) for the *gltA*, *ftsZ*, and *groEL* sequences of *Bartonella* strains, and the *IS1111* sequence for *Coxiella burnetii* as previously described [11]. The diagnosis of IE was confirmed for *Bartonella henselae* and *C. burnetii* by the presence of IgG antibodies ≥ 800 and/or a positive result of molecular tests in blood samples and heart valves.

### 2.3. Statistical Analysis

In-hospital mortality was defined as all in-hospital deaths due to IE. Data concerning age, sex, pre-existing conditions, vegetation, type of valve, source, surgery, and microorganism information were included. A descriptive analysis of the variables was performed, quantitative variables are presented as median (1st–3rd quartile), and categorical variables are presented as absolute and relative frequencies The Mann-Whitney U test, chi-square test, and Fisher’s exact test were used to compare differences between groups, as appropriate. The Mann-Kendall test was used to evaluate the temporal trend of a variable. Bivariate analyses and stepwise multivariate logistic regression with backward elimination were used to identify risk factors independently associated with in-hospital mortality, and the results are presented as relative risk (RR) and 95% confidence intervals. The normality of continuous variables was tested. Poisson regression analysis was performed for trend tests. Univariate and then stepwise logistic regression analyses were performed to determine independent predictors of in-hospital mortality. The multivariate Cox proportional hazards model was used to estimate the hazard ratio (HR) of predictors of in-hospital mortality. The proportional hazard assumption was tested and found to be valid by inspection of log-log plots, scaled Schoenfeld residuals, and tests of the nonzero slope. The Kaplan-Meier method was used to generate survival curves, and survival rates between groups were compared using the log-rank test. All analyses were performed using STATA^®^ version 15.0 (StataCorp LP, College Station, TX, USA), and statistical significance was defined as *p* < 0.05.

### 2.4. Ethics Statement

This study was conducted in accordance with the principles of the Declaration of Helsinki and approved by the Research Ethics Committees of HUPE (CAAE- 01247512.3.0000.5259), HUCCF (CEP- No 517/01), and FIOCRUZ (CAAE- 39056120.6.0000.5248).

## 3. Results

### 3.1. Baseline Characteristics of Patients with Infective Endocarditis

Between January 2009 and June 2021, a total of 251 patients with IE were hospitalized in both institutions, and 11 patients were excluded from the analysis because of transfer to another hospital or missing data. The median age of the cohort was 55 years, 57% were male, and 41% of IE patients had pre-existing conditions especially chronic kidney disease and valvular heart disease. No patients had a history of intravenous drug use. Infective endocarditis most often affected the native valve and occurred on the left side in 75% of cases. However, IE involving a prosthetic valve occurred more frequently in patients aged ≥ 60 years with an increasing trend in the proportion of prosthetic valves affected by IE per year. The vegetation was most frequently located on the mitral valve, followed by the aortic, tricuspid, and pulmonary valves. In addition, involvement of more than one valve was identified in 13% of patients, mainly aortic/mitral valves. Healthcare-associated infective endocarditis was the most frequently reported classified into dialysis-associated HAIE (44%) and non-dialysis-associated HAIE. Poisson regression analysis did not show any trend in the annual proportion of HAIE. During the study period, the surgery rate was 29%, and surgical treatment was significantly more frequent in left-sided IE. Patients who underwent surgical treatment had significantly longer hospital stays than those who received medical treatment. The detailed findings of the patients with IE are summarized in Table 1.

### 3.2. Etiology

The main microorganisms identified in blood samples were *Staphylococcus aureus* (26%), *Streptococcus* spp. (21%), and *Enterococcus* spp. (18%). Fastidious growing bacteria were identified using immunological and molecular methods in 3% (*Bartonella* spp., *C. burnetii*, HACEK group, *Abiotrofia defective*, *Gemella morbillorum,* and *Microbacterium testaceum*). Finally, blood cultures were negative in 19% of cases (Table 2).

*Staphylococcus aureus* (35%) and *Enterococcus* spp. (25%) were significantly more frequently identified in HAIE, whereas the viridans group streptococci (26%) and *Streptococcus bovis* group (10%) were significantly more frequently identified in CAIE (Table 3).

### 3.3. Mortality Analysis

**Figure 1:**
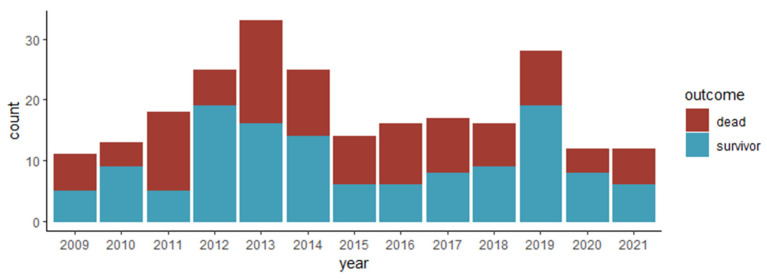
Distribution of infective endocarditis cases by year and outcome.

Overall in-hospital mortality was 45.8%. The mortality was significantly higher in patients aged ≥ 60 years (53%), CCI score ≥ 3 (60%), HAIE (53%), left-sided IE (51%), and enterococcal IE (67%). Poisson regression analysis revealed no trend in hospital mortality per year (Figure 1).

**Figure 2:**
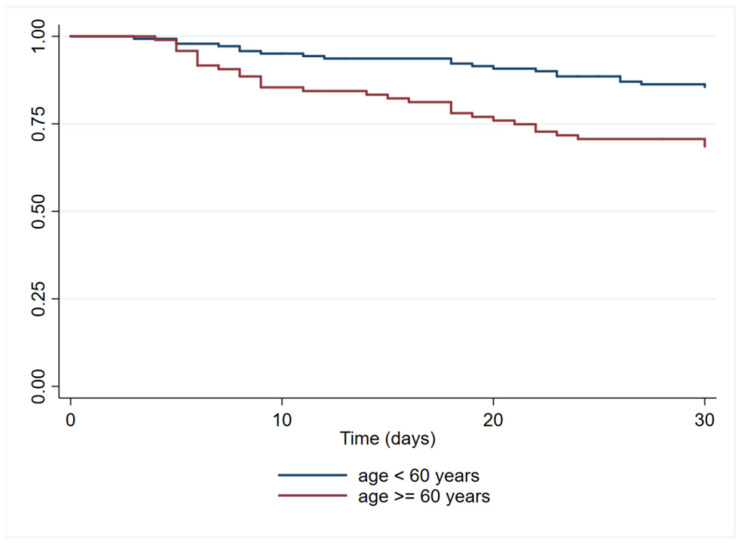
Patients aged ≥ 60 years had higher in-hospital mortality (HR = 1.9; 95% CI 1.2–3.1; *p* = 0.008).

In the univariate analysis, age ≥ 60 years, CCI score ≥ 3, left-side IE, HAIE, and enterococcal IE were significantly and positively associated with in-hospital mortality (Table 1). The variables that were included in the univariate and multivariate Cox regression models for in-hospital mortality are shown in Table 4. Finally, it was determined that only age ≥ 60 years was an independent risk factor for mortality in patients with IE in the adjusted model (HR = 1.9; 95% CI 1.2–3.1; *p* = 0.008, Figure 2).

## 4. Discussion

Although substantial advances have been made in the diagnosis and treatment of patients with IE, morbidity and mortality rates remain very high, especially in developing countries. The present retrospective cohort study assessed the clinical and laboratory characteristics of patients with IE in two tertiary centers in Rio de Janeiro between January 2009 and June 2021. The higher mortality rate in patients over 60 years of age is in accordance with the findings in a study published by Chen et al. [14] on the worldwide burden and trends of IE from 1990 to 2019. The results showed an increasing trend in almost all countries during the study period, mainly affecting men ≥ 50 years of age, demonstrating the potential influence of age on IE morbidity. Among them, the estimated annual incidence increased by 1.2% per year, whereas the mortality was 0.71%. Thus, the incidence and mortality from IE in patients aged > 50 years increased from 35% and 63% in 1990 to 60% and 79% in 2019, respectively [14]. The increasing trend in the incidence of IE in older people is associated with an aging population and the increase in the proportion of older people [15]. In addition, studies have shown an increase in the incidence of prosthetic valve endocarditis in recent years, mainly in older adults, as evidenced in this study [16].

Invasive interventions are often performed in older adults. These interventions including electronic cardiovascular devices and invasive diagnostic, or therapeutic procedures are independent predictors of mortality [15,17].

In our retrospective cohort of definite cases of IE in two university hospitals in Rio de Janeiro, patients aged ≥ 60 years had a more frequent history of diabetes and use of intracardiac devices and prosthetic valves than younger patients. In our previous investigations [9,18] in which IE in low- and middle-income countries was reviewed, we had access to only 12 studies that included data from multivariate analyses of in-hospital mortality rates in IE patients. These studies showed statistically significant relationships between age > 45 years and chronic renal failure, septic shock, heart failure, prosthetic dysfunction, nosocomial IE, neoplasia, mobile vegetation, mental alterations, central nervous system emboli, coronary artery disease, aortic vegetation, and large vegetation. The in-hospital mortality in South America ranges from 24.0% to 46.4% [4,8,11]. Furthermore, it is necessary to consider an accelerated aging rate in the Brazilian population [19], which could suggest increasing comorbidities and, consequently, the risk of IE.

There is evidence of an increase in the incidence of HAIE worldwide [9,18]. This study did not show an increase in HAIE, but the proportion continues to be similar to that reported in a previous study (56.3%) [20]. This leads to an academic reflection on the need for greater efforts to prevent HAIE and reduce the prevalence of endocarditis. Although evidence suggests that early surgery can improve survival in patients with complicated IE, this remains controversial [21]. In this study, despite a significant increase in the rate of surgery, no improvement in survival was observed, similar to that in other cohort studies [18,22].

In this Rio de Janeiro cohort of infective endocarditis, the vegetation was most frequently located on the mitral valve (43%) and other locations were aortic (22%), tricuspid (11%), and pulmonary (1%). In addition, involvement of more than one valve was identified in 13% of patients, mainly aortic/mitral valves. According to the literature, more than 50% of vegetation generally affects the left side of the heart and is most frequently associated with mitral valve prolapse and mitral and aortic degenerative lesions [13]. Infective endocarditis often represents a condition that is difficult to diagnose, especially in the initial phase of the illness, due to its indistinct clinical manifestations. Thus, patients with fever and predisposing factors must undergo echocardiography, which is an essential method for the diagnostic evaluation of patients suspected of having IE.

Regarding the etiology of IE, no significant variation has been observed in the frequency of microorganisms since 2009 [20]. Thus, *S. aureus* (26%), *Streptococcus* spp. (21%) and *Enterococcus* spp. (18%) continue to be the most frequently identified microorganisms. Recent studies worldwide coincide with *S. aureus* and *Streptococcus* spp. as the most frequent etiologies of IE. However, an increase in the incidence of IE due to *Enterococcus* spp. has been reported, and the factors involved must be studied [23,24]. In addition, the highest in-hospital mortality was found in cases of IE due to *Enterococcus* spp. (11.7%) and *S. aureus* (10.4%), similar to what has been shown in recent studies [25,26]. The *Enterobacteriaceae* family is a rare cause of IE, and it was identified in 9% of patients in this cohort. In a previous publication, it was shown that the molecular characteristics of *Escherichia coli* and *Klebsiella pneumoniae* isolated from the blood of patients with IE were similar to those isolated from urine; therefore, urinary tract infections should be monitored as a source of bacteremia and IE [27].

Blood cultures were negative in 19% of cases, corresponding to the highest proportion of the group of patients with CAIE (24%) compared to HAIE (14%). The percentage of patients with IE with negative blood cultures may vary depending on the availability of immunological and molecular methods that enable the identification of infectious agents such as *C. burnetii,* a major criterion for diagnosis, or the genus *Bartonella,* a minor criterion in the diagnosis of IE. In this study, *B. henselae* and *C. burnetii* were identified in 0.8% and 0.4% of the cases, respectively. In our first report, we did not have the opportunity to identify these etiologies [28]. However, in recent years, our research team has been able to advance the investigation of patients with negative blood cultures due to the cooperation of the Fundação Oswaldo Cruz, Rio de Janeiro, Brazil. Therefore, the reference laboratory performed diagnostic analyses using indirect immunofluorescence assays and molecular tests. Nevertheless, it is also necessary to consider the previous use of antibiotics prior to blood culture is the main cause of blood culture-negative infective endocarditis [4].

Regarding the in-hospital mortality of IE, the study showed that overall, in-hospital mortality was 45.8%, similar to that of another study conducted in Brazil [29]. Independent predictors of mortality were age > 60 years, left-sided IE, and HAIE. Despite significant advances in recent decades, IE is still associated with high mortality, i.e., 25 to 30% in six months [30], and elderly patients are at a higher risk of in-hospital mortality and complications after surgery than younger patients [31]. Nevertheless, due to the lack of large multicenter cohorts and the heterogeneity of this population, there is insufficient evidence to support specific recommendations in management guidelines [32]. In this study, age > 60 years increased the risk of death almost twofold.

A recent, elegant study developing in Romania [33], where 92 patients with definite IE participated, from January 2010 until December 2019, showed the mean age of patients with definite IE was 63.8 years, half of them being older than 65 years, and 49 (53.3%) patients were male, death occurred in 31 (33.7%) patients. On the other hand, in Rio de Janeiro, Brazil, in the last 13 years, after diagnosing 240 patients with definite IE, we observed a median age of 55 years (IQR: 39–66 years), 136 (57%) were male and 110 (45.8%) patients did not survive. Justifying this moment we have a trend of older men with IE and the mortality rate is still very high, particularly in Brazil.

This study has some limitations. First, due to its retrospective design, there is information bias regarding medical and surgical treatments, which limits its analysis. Second, there is the possibility of unrecorded confounding factors such as socioeconomic variables and living conditions, which may have affected the results. Third, there was no information on long-term survival or the occurrence of additional complications after hospital discharge, which may have resulted in an underestimation of mortality. Fourth, this was not a population-based study, and mortality rates could not be calculated in comparison to other studies. Finally, our study was conducted exclusively in Rio de Janeiro, and generalization to other regions of the country is not guaranteed. However, an advantage could be that two important tertiary hospitals were included which both had a team that evaluated these patients during the study period. The findings of this study also allowed the identification of other infectious bacterial agents, mainly the HACEK group of fastidious bacteria, in addition to fungi such as *Candida* spp. and the emerging opportunistic fungal pathogen *Rhodotorula mucilaginosa*.

## 5. Conclusions

This study presents a mortality analysis of a 12-year cohort of patients with IE in the metropolitan area of Rio de Janeiro, Brazil. Overall, in-hospital mortality was 45.8%, and mortality was significantly higher in the following patients: age ≥ 60 years (53%), CCI score ≥ 3 (60%), HAIE (53%), left-sided IE (51%), and enterococcal IE (67%). There was no evidence of any significant trend in mortality from IE, and age ≥ 60 years was an independent risk factor for in-hospital mortality. Thus, a consensus is required regarding the management of IE in this group of patients. Finally, we highlight the need to include immunological and molecular methods for the etiological diagnosis of fastidious agents that require special techniques for their isolation.

## Figures and Tables

**Table 1:** Baseline characteristics of patients with infective endocarditis in Rio de Janeiro, January 2009 to June 2021.

	Total *n* = 240		Survivor *n* = 130		Dead *n* = 110		*p*
**Age** (years) *	55	(39–66)	50	(32–60)	61	(48–71)	**<0.001 ^‡^**
≥60 years	97	41%	36	28%	61	55%	**<0.001** ^¶^
**Sex**							
Female	104	43%	55	42%	49	45%	0.727 ^¶^
Male	136	57%	75	58%	61	55%	
**Pre-existing conditions**							
Charlson comorbidity index ≥ 3	99	41%	40	31%	59	54%	**<0.001** ^¶^
Chronic kidney disease	77	32%	36	28%	41	37%	0.113 ^¶^
Valvular heart disease	76	32%	40	31%	36	33%	0.745 ^¶^
History of dialysis	60	25%	28	22%	32	29%	0.178 ^¶^
Diabetes	50	21%	26	20%	24	22%	0.730 ^¶^
Congenital heart disease	25	10%	18	14%	7	6%	0.059 ^¶^
Previous infective endocarditis	18	8%	6	5%	12	11%	0.065 ^¶^
Cancer	17	7%	9	7%	8	7%	0.916 ^¶^
HIV infection	13	5%	7	5%	6	5%	0.981 ^¶^
Intracardiac device	12	5%	5	4%	7	6%	0.373 ^¶^
**Vegetation ^†^**							
mitral	102	43%	50	38%	52	47%	0.169 ^¶^
aortic	52	22%	25	19%	27	25%	0.319 ^¶^
tricuspid	27	11%	20	15%	7	6%	**0.028** ^¶^
other location	14	6%	7	5%	7	6%	0.734 ^¶^
left-sided	180	75%	88	68%	92	84%	**0.004** ^¶^
**Type of valve**							
native	186	77.5%	106	82%	80	73%	0.103 ^¶^
prosthetic	54	22.5%	24	18%	30	27%	
**Source**							
Community-associated IE (CAIE)	111	46%	69	53%	42	38%	
Healthcare-associated IE (HAIE)	129	54%	61	47%	68	62%	**0.021** ^¶^
**Surgery**							
Surgical treatment	69	29%	36	28%	33	30%	0.694 ^¶^
Number of days of hospitalization *	45	(28–78)	54	(36–84)	34	(18–61)	**<0.001** ^§^
Number of days until surgery *	21	(–35)	17	(6–35)	25	(10–35)	0.314 ^§^

* Median (1st–3rd quartile), ^†^ Some patients had lesions in more than one place ^‡^ Student’s *t*-test, ^§^ Mann-Whitney U test, ^¶^ Chi square test. Bold type is the significant *p* value.

**Table 2:** Microbiological profile of patients with infective endocarditis in Rio de Janeiro, January 2009 to June 2021.

Microorganism	Total *n* = 240		Survivor *n* = 130		Dead *n* = 110		*p*
*Staphylococcus aureus*	62	26%	37	29%	25	23%	0.263 *
MRSA *	19	8%	11	9%	8	7%	0.695 *
*Streptococcus* spp.	49	21%	34	27%	15	14%	0.013 *
Viridans group streptococci	29	12%	21	17%	8	7%	0.030 *
*Streptococcus bovis* group	12	5%	8	6%	4	4%	0.391 ^†^
*Enterococcus* spp.	42	18%	14	11%	28	25%	0.004 *
*Enterococcus faecalis*	33	14%	10	8%	23	21%	0.004 *
VRE ^†^	4	4%	1	2%	3	6%	0.658 ^†^
Coagulase negative *Staphylococci*	21	9%	10	8%	11	10%	0.556 *
*Enterobacteriaceae*	11	5%	6	5%	5	5%	0.948 *
Fastidious bacteria	8	3%	5	4%	3	3%	0.728 ^†^
*Bartonella henselae*	2	0.8%	2	1.5%	0	0%	
*HACEK* group	2	0.8%	2	1.5%	0	0%	
*Abiotrofia defective*	1	0.4%	0	0%	1	0.9%	
*Coxiella burnetii*	1	0.4%	1	0.8%	0	0%	
*Gemella morbillorum*	1	0.4%	0	0%	1	0.9%	
*Microbacterium testaceum*	1	0.4%	0	0%	1	0.9%	
Fungus	8	3.4%	4	3.2%	4	3.6%	1.000 ^†^
*Candida* spp.	7	3.0%	3	2.4%	4	3.6%	
*Rhodotorula mucilaginosa*	1	0.4%	1	0.8%	0	0%	
Negative	45	19%	25	19%	20	18%	0.836 *

Abbreviations: MRSA, Methicillin-resistant *Staphylococcus aureus*; VRE, Vancomycin-resistant *Enterococcus*. * Chi square test, ^†^ Fisher exact test. Bold type is the significant *p* value.

**Table 3:** Microbiological profile by source of infection.

Microorganism	Total *n* = 240		CAIE *n* = 111		HAIE *n* = 129		*p*
*Staphylococcus aureus*	62	26%	17	16%	45	35%	0.001 *
*Streptococcus* spp.	49	21%	43	39%	6	5%	<0.001 *
Viridans group streptococci	29	12%	28	26%	1	1%	**<0.001** ^†^
*Streptococcus bovis* group	12	5%	11	10%	1	1%	**0.002** ^†^
*Enterococcus* spp.	42	18%	10	9%	32	25%	0.001 *
*Enterococcus faecalis*	33	14%	9	8%	24	19%	**0.020** ^†^
Coagulase negative *staphylococci*	21	9%	10	9%	11	9%	0.875 *
*Enterobacteriaceae*	11	5%	2	2%	9	7%	0.069 ^†^
Fastidious bacteria	8	3%	6	6%	2	2%	0.148 ^†^
Fungus	8	3%	1	1%	7	5%	0.073 ^†^
Negative	45	19%	23	21%	16	13%	0.075 *

Abbreviations: MRSA, Methicillin-resistant *Staphylococcus aureus*; VRE, Vancomycin-resistant *Enterococcus*. * Chi square test, ^†^ Fisher exact test. Bold typing is the significant *p* value.

**Table 4:** Multivariable Cox proportional hazard ratios for patients with infective endocarditis in Rio de Janeiro, January 2009 to June 2021.

Variable	Unadjusted HR (95% CI)		*p*	Adjusted HR (95% CI)		*p*
Age ≥ 60 years	2.0	(1.4–2.9)	**0.000**	1.9	(1.2–3.1)	**0.008**
CCI score ≥ 3	1.6	(1.1–2.4)	**0.016**	1.1	(0.6–1.7)	0.815
Left-side IE	1.5	(0.9–2.6)	**0.036**	1.5	(0.9–2.5)	0.121
HAIE	1.0	(0.7–1.5)	0.996	1.1	(0.7–1.6)	0.789
Enterococcal IE	1.2	(0.8–1.9)	0.357	1.0	(0.7–1.6)	0.902

Abbreviations: CCI, Charlson comorbidity index; IE, infective endocarditis; HAIE, Healthcare-associated infective endocarditis; HR, hazard ratio. Bold type is the significant *p* value.

## Data Availability

Data are contained within the article.
